# Cardiac Manifestations of Antiphospholipid Syndrome With Focus on Its Primary Form

**DOI:** 10.3389/fimmu.2019.00941

**Published:** 2019-05-10

**Authors:** Tamara Kolitz, Shachaf Shiber, Itzhak Sharabi, Asher Winder, Gisele Zandman-Goddard

**Affiliations:** ^1^Department of Medicine C, Wolfson Medical Center, Holon, Israel; ^2^Department of Rheumatology, Rabin Medical Center, Petach Tikva, Israel; ^3^Sackler Faculty of Medicine, Tel Aviv University, Tel Aviv, Israel; ^4^Department of Cardiology, Wolfson Medical Center, Holon, Israel; ^5^Department of Hematology, Wolfson Medical Center, Holon, Israel

**Keywords:** primary antiphospholipid syndrome, secondary antiphospholipid syndrome, APS antiphospholipid antibodies, cardiac manifestations, cardiovascular disease, heart valve disease, myocardial infarction, pulmonary hypertension

## Abstract

Antiphospholipid syndrome (APS) is a multisystem autoimmune disease most commonly associated with recurrent arterial and venous thromboembolism and recurrent fetal loss. Other possible antiphospholipid antibody (aPL)-related clinical manifestations include cardiac involvement. The heart can be involved through immune mediated and /or thrombotic mechanisms. Mortality due to cardiovascular problems is elevated in APS. However, the cardiovascular risk in patients with primary APS (PAPS) compared with lupus-related APS is yet to be established. Cardiac symptoms of APS include valve abnormalities (thickening and vegetations), coronary artery disease (CAD), myocardial dysfunction, pulmonary hypertension, and intracardiac thrombi. Heart valve lesions are the most common cardiac manifestation, observed in approximately one third of PAPS patients and usually do not cause hemodynamic significance. Deposits of immunoglobulins including anticardiolipin (aCL), and of complement components, are commonly observed in affected heart valves from these patients. This suggests that an inflammatory process is initiated by aPL deposition, eventually resulting in the formation of valvular lesion. aPL may have a direct role in the atherosclerotic process via induction of endothelial activation. Multiple traditional and autoimmune-inflammatory risk factors are involved in triggering an expedited atherosclerotic arterial disease evident in APS. It is imperative to increase the efforts in early diagnosis, control of risk factors and close follow-up, in the attempt to minimize cardiovascular risk in APS. Clinicians should bear in mind that a multidisciplinary therapeutic approach is of paramount importance in these patients. This article reviews the cardiac detriments of APS, including treatment recommendations for each cardiac complication.

## Introduction

The antiphospholipid syndrome (APS) is an autoimmune disease characterized by the presence of antiphospholipid antibodies (aPL) leading to arterial and venous thrombosis and pregnancy morbidities (recurrent fetal loss and placental insufficiency) (1). The most frequently found subgroups of aPL are Lupus anticoagulant (LAC), anticardiolipin antibodies (aCL), and anti-β_2_-glycoprotein I (β_2_ GPI) antibodies. aPL are not only diagnostic but also pathogenic autoantibodies. The presence of aPL, though necessary, is not sufficient by itself to induce clotting, therefore an involvement of an additional trigger is expected. The most common second trigger identified is the inflammation secondary to infectious agents (2).

APS was initially considered as an autoimmune coagulopathy, but the extensive accumulated data has clarified that it is in fact a complex and systemic autoimmune disease. APS occurs as a primary disorder (primary APS, PAPS) or as a detrimental manifestation secondary to another autoimmune disease (secondary APS, SAPS), most commonly systemic lupus erythematosus **(**SLE). The annual risk of thrombosis among individuals with aPL in the setting of SLE is likely < 4 percent, and the risk in individuals with aPL without SLE is likely < 1 percent (3).

Due to its vascular nature, various organs and tissues may be affected, including the cardiac system. The cardiac involvement in APS is multifactorial: thrombosis plays an important role as well as immune-mediated injury (4, 5). The most common cardiac manifestations are valvulopathies, ranging from valve thickening through non-bacterial thrombotic endocarditis (NBTE; Libman-Sacks endocarditis) to regurgitation and severe valvular damage, and coronary artery disease (CAD). Valvulopathies and CAD are the main cardiac manifestations in APS, while other less common cardiac manifestations include myocardial dysfunction, pulmonary hypertension and intracardiac thrombus. As expected, older age is yet another risk factor for occurrence of overall cardiac manifestations in PAPS patients (6). Similarly to treatment of other aspects of APS, the cornerstone of therapy in most APS-related cardiac manifestations is anticoagulation.

This article will review the cardiac involvement in APS, including criteria and non-criteria cardiac manifestations and treatment recommendations, with a focus on PAPS (Table 1).

**Table 1:** Summary of cardiac involvement in APS and treatment recommendations.

**Cardiac abnormality**	**Prevalence—PAPS**	**Prevalence—SAPS**	**Recommended management for PAPS patients**
***Coronary artery disease***			1. *Aggressive control of cardiovascular risk factors* 2. *Consider statins, HCQ* 3. *In the presence of thrombosis - anticoagulation* 4. *Consider PCI and/or CABG*
*Asymptomatic atherosclerosis*	~15%	~30–35%	
Myocardial infarction	1.2%^*^	3.8%^*^ (in APS-SLE)	
***Valvular disease***	~33%^**^	~40–50%^**^	1. *TTE for every APS patient as initial screening* 2. *Asymptomatic patients: prophylactic low-dose aspirin* 2. *Symptomatic patients: stroke prophylaxis with anticoagulation*, 3. *Consider Surgical consultation*
Non-bacterial thrombotic vegetations		
Valvular fibrosis and thickening		
Valvular regurgitation		
***Myocardial dysfunction***	*No reliable data*	*No reliable data*	1. *Anticoagulation* 2. *Standard therapy for systolic heart failure*
Diffuse cardiomyopathy		
Diastolic dysfunction		
***Intracardiac thrombosis***	*No reliable data*	*No reliable data*	1. *Intense Anticoagulation (target INR 3–4)* 2. *Cardiac surgeon consultation*
***Pulmonary hypertension***	1–5.7%^**^	0.5–14%^**^	1. *Anticoagulation* 2. *Surgical thromboendarterectomy*

* *According to the Euro-phospholipid cohort, 10 year follow-up (7)*.

** *When assessed by TTE*.

## Classification Criteria and Non-criteria Manifestations in APS

The revised Sapporo APS Classification Criteria defines that APS is present in patients who match at least one clinical and at least one laboratory criteria (1). Classification criteria are an important tool for appropriate inclusion of homogeneous groups of patients in observational and therapeutic studies. A number of other features associated with APS can be exhibited, but these are termed “non-criteria” because they are not included in the revised classification criteria. In addition, non-criteria only, even when combined with persistent aPL, does not provide a definite diagnosis of APS. However, the recent consensus conference in Australia accepted heart valve disease (HVD) as an integral manifestation of APS, yet not as a diagnostic criterion (1).

Even though there are classification criteria for APS and for SLE, it is challenging to determine between the two since they share some manifestations. A few articles brought up the question of whether APS and SLE are distinct diseases or not. Some authors concluded that these two autoimmune diseases are part of a spectrum ranging from PAPS only through overlap SLE-APS to aPL-negative-SLE (8). Approximately 40% of SLE patients have aPL but about one third of them will eventually develop clinical events (9). The incidence of also developing SLE is reported in 4–10% of PAPS patients (10), and the switch can occur many years after the initial diagnosis has been established (7). A 10 year-follow-up in the Euro-Phospholipid project revealed that eight patients initially diagnosed with PAPS, developed elevated titers of anti-dsDNA antibodies and were diagnosed as lupus-like syndrome. Three additional patients were diagnosed as overlap SLE-APS (11). It is of major importance for clinicians to define which patients are at higher risk for developing full blown SLE, and closely follow-up these patients (12). A recent review found that 28% of PAPS patients could have been mistakenly classified as SLE based upon the Systemic Lupus Erythematosus International Collaborating Clinics (SLICC) criteria, and were thus subjected to inappropriate hazardous treatments. None of these PAPS patients developed SLE during the median follow-up period of 12 years. The authors thus emphasized the need to develop classification criteria for PAPS that will enable distinguishing it from SAPS (12).

## Types of Cardiac Involvement in APS

### Heart Valve Disease (HVD)

HVD in APS patients is defined by the presence of valve lesions and/or moderate to severe valve dysfunction in the absence of a history of rheumatic fever or infective endocarditis (1). Valvular involvement was the earliest reported cardiac manifestation of APS (13) and is the most common cardiac manifestation of APS. It includes valvular thickening and valve vegetations (also referred as Libman-Sacks endocarditis) (14). Valve lesions in APS are characterized by localized valve thickness >3 mm involving the proximal or middle portion of the leaflets and irregular nodules on the atrial area of the mitral valve or the vascular side of the aortic valve are typical. The most commonly affected valves are the mitral valve, followed by the aortic valve (15, 16). In general, different studies have shown that approximately one third of PAPS patients have HVD when evaluated by transthoracic echocardiography (TTE), and that the prevalence was significantly higher compared with healthy individuals in whom valve lesions were evident in 0 to 5 percent (14, 17–23). However, the prevalence of HVD in PAPS ranges from 10% to more than 60% in various studies (24). Due to the different imaging techniques (transthoracic [TTE] or transesophageal [TEE] echocardiography) utilized for assessing HVD. Another important point is the comparison of HVD prevalence in PAPS and SLE-associated APS. Only few studies have directly compared these two groups. It was shown that the average prevalence of valve involvement was found to be significantly higher in the secondary APS group (41 vs. 27%, *P* = 0.007) (25, 26). In parallel, SLE patients who are aPL-positive compared to aPL- negative exhibit a significantly greater prevalence of valvular abnormalities (27). In a meta-analysis of aPL-associated HVD in SLE patients, the risk of HVD was higher in patients with LAC or IgG aCL (OR 6) compared with IgM aCL (OR 3) (23). Moreover, nearly 90% of SLE patients with HVD had positive aPL compared to 44% of SLE patients without HVD. Valve impairment in PAPS is usually asymptomatic but it can lead to significant dysfunction. A progression to severe valvular regurgitation requiring surgery is found in ~4–6% of APS patients with HVD (15). A history of arterial thrombosis increases the co-morbidity of HVD in APS patients (51%) compared with a history of venous thrombosis (42%) (20, 21, 28, 29). Aortic valve lesions, confer an increased risk of stroke (27). A retrospective study of 284 APS patients, 159 of whom diagnosed with PAPS, found significant correlations between HVD and CNS manifestations (epilepsy, migraines, cerebrovascular accidents, and transient ischemic attacks), while patients with SAPS had no such correlations. Thus, CNS manifestations in PAPS may occur in the presence of valvulopathy (25).

The histopathological characteristics of aPL-associated valvulopathy are non-specific and include fibrosis, calcification, vascular proliferation, verrucous thrombosis on endocardial valvular surfaces, and thrombosis of intravalvular capillaries (19, 28, 30). Microscopy of deformed heart valves from APS patients revealed depositions of immunoglobulins including aCL (mainly IgG) and complement components, located along the exterior of the leaflets and cusps. Such deposits were not evident in control valves from aPL-negative patients and APS patients without valve involvement (31). Possibly, aPL may cause a sub-endocardial inflammatory process through an interaction with antigens on valve surfaces (32). The mechanisms of thrombosis and inflammation subsequently lead to fibrosis and calcification and eventually to valve deformation (19). There is some evidence in the literature of an association between the valve lesions in rheumatic fever (RF) and the presence of aPL. A study which evaluated the serum of 90 patients with RF and of 42 patients with APS, showed that 24% of patients with rheumatic heart disease had positivity for anti-β_2_GPI and that patients with APS had anti-streptococcal activity, recognizing the M protein in 16.6% of cases. The authors concluded that there is considerable overlap of humoral immunity, supporting the hypothesis of common pathogenic mechanisms in development of valvular manifestations in APS and RF (33). Another work showed that 80% of RF patients were positive for aCL antibodies during active phase of the disease (34). More studies are needed to evaluate a potential association between RF-associated valve lesions in RF and the presence of aPL.

### Coronary Artery Disease

#### Atherosclerosis

The pathogenesis of premature accelerated atherosclerosis has been assessed in APS patients (35), despite a similar incidence of traditional Framingham risk factors in APS patients compared to the general population (5). Evaluation of subclinical atherosclerosis of the carotid and femoral (by ultrasound [US]) in 86 patients with primary APS or SLE/APS compared to patients with diabetes mellitus and healthy controls revealed 28% of APS patients had carotid atherosclerotic plaques compared to 23% of SLE/APS patients and 30% of patients with diabetes mellitus (36). The relative risk for cardiovascular disease was 2.5. In SLE patients who are aPL positive, plaques were observed in 6–31% of patients (36). Another study included 197 SLE patients and 197 matched controls who underwent carotid US. Atherosclerotic plaques were observed in 37.1% of the SLE population vs. 15.2% of controls (*p* < 0.001). The prevalence of any aPL was not significantly different between SLE patients with or without a carotid plaque. However, aCL antibodies were significantly more common in patients with no plaque compared to those with a plaque—thus, no correlation was found between aCL antibodies and carotid atherosclerosis (37). Atherosclerosis leading to major cardiovascular morbidity and mortality remains an important concern, predominantly in the younger population (5, 38). Other methods to detect subclinical atherosclerosis include evaluation of early endothelial dysfunction, circulation impairment and atherosclerotic plaques (36). However, studies, assessing the presence of an accelerated atherosclerosis in PAPS patients, were inconclusive. Discrepancies were demonstrated with respect to the number of atherosclerotic plaques in the carotid arteries by measurements of the intima media thickness (IMT), which is an early sensitive marker of generalized atherosclerosis (38, 39). Among 28 PAPS patients, an increased prevalence of carotid artery plaques by IMT measurements and a reduced lumen diameter were demonstrated and could be a significant risk for stroke (38). Contrary to these results, another study found similar results on IMT in PAPS patients without CAD risk as compared to controls, and therefore concluded that PAPS itself may not contribute to the risk of premature atherosclerosis. The authors suggested that previous reports that identified endothelial dysfunction by IMT in PAPS, did not exclude CAD risk or aging, which may explain their different findings (40).

The various types of aPL may play different roles in thrombosis formation and atherogenesis. In a retrospective study of 1,519 aPL-positive individuals, 637 patients were considered to have APS. Venous thrombotic events prevailed in patients with LAC, while peripheral, coronary and carotid thrombosis were more frequent in patients with significantly elevated titers of IgG or IgM aCL (41). A study which evaluated whether the presence of aPL was in any way associated with the IMT of carotid arteries demonstrated that IMT values in all tested carotid segments could be independently predicted by the IgG-aCL titer (42). This study further supports the link between the accelerated atherosclerotic process and aPL, specifically aCL and its cofactor, β_2_GPI in these patients (42–44). Premenopausal women with APS were compared with age-matched groups of patients with SLE or rheumatoid arthritis and healthy participants for cardiovascular risk factors and early atherosclerosis. Among premenopausal women with APS, carotid, and femoral plaques were more frequently observed in the absence of other atherosclerotic risks. However, in contrast to the previously mentioned studies, this study could not establish a connection between aCL or anti-β_2_GPI antibodies and atherosclerosis (45). Similar results on the lack of association between aCL and anti-β2GPI antibodies and expedited atherosclerosis were revealed in a cohort of rheumatologic patients who underwent coronary bypass (46).

It has been suggested that the pro-inflammatory and pro-coagulant activity of aPL on vascular endothelial cells might be directly related to atherosclerosis in PAPS. The indirect impact through inflammatory and immune mechanisms was also proposed. These mechanisms are assumed to induce autoantibody-mediated thrombosis (5, 40, 41) through an autoantibody cross-reaction (5), by oxidative stress (47–50). *In vitro* studies from patients with primary or secondary APS depict β2GPI that binds to oxidated low-density lipoprotein (LDL), but not to native LDL. This non-dissociable complex formation is associated with the process of autoimmune-mediated atherogenesis (49, 51). Different studies have confirmed the presence of autoantibodies directed against serum lipoproteins; a high percentage of these antibodies also cross-react (52, 53).

#### Myocardial Infarction

The occurrence of acute myocardial infarction (AMI) in APS has been known since the syndrome was first described (54). CAD may be the first presentation of APS (55). The association between aPL and AMI is more frequent in women (56). There is some evidence that ~2.8–5.5% of cases with AMI in young individuals are secondary to APS. AMI in patients with APS usually occurs in the fourth decade of life (57). MI in the young is usually associated with normal or nearly normal coronary angiographic studies (56, 58). In the Euro-phospholipid cohort, which contains the largest series of 1,000 APS patients, there were significantly more MI events in the APS-SLE group (3.8%) compared to the PAPS group (1.2%) over a 10-year follow up period. In regard to the causes of death depending on the underlying disease, 10% of PAPS patients died of MI in contrast to no patients in the SLE group, but without statistical significance of the underlying disease (PAPS or SAPS) (11). A recent systematic review included 40 patients with acute MI secondary to APS, of whom 33 patients were classified as PAPS. MI was the first presentation of APS in 80% of these cases. Cardiac catheterization revealed normal coronaries in 75% of patients, whereas 25% had evidence of obstructive atherosclerotic stenosis (59).

Accelerated atherosclerosis of the coronary arteries as well as microvascular injury or coronary thromboembolism may lead to ischemia. Coronary embolism is presented most commonly as one of the major causes of MI in the young (60). A strong correlation between thrombophilic conditions and MI in APS patients exists, but not for other thrombophilic disorders (61). LV regional wall motion abnormalities (WMA) correlated with high titers of aPL (62). The LV WMA might be related to MI as a result of CAD, or microvascular injury leading to myocardial necrosis. The specific role of each aPL subtype in APS is yet to be elucidated. In a study of 214 young patients, titers of five criteria and non-criteria aPL were measured. These included aCL anti-phosphatidylserine (aPS), anti-phosphatidylinositol (aPI), anti-phosphatidylcholine (aPC), and anti-phosphatidylethanol amine (aPEA). The titers of aPL in patients with MI were significantly elevated in the younger age group under 50 years old (63). The presence of elevated aCL antibody titers was found to be an independent risk factor for MI or cardiac death in a cohort of middle aged dyslipidemic men (64). Several studies have found significant correlations between aCL and anti-β2GPI levels and the incidence and severity of acute coronary syndrome (ACS). An association between elevated titers of anti- β2GPI antibodies and a significant risk for MI in young menopausal women was evaluated in 172 patients hospitalized for a first MI before the age of 45 years and compared to 172 matched controls. The contribution of elevated anti- β2GPI antibody titers and the risk of MI was independent of other risk factors, including coronary atherosclerosis (36, 65). In a prospective study of CAD and stroke in 8006 American men, elevated titers of aCL antibodies as a risk factor for MI was examined by using stored frozen sera obtained from subjects enrolled in the study and followed up for 20 years. The results suggested that β2GPI-dependent aCL of the IgG class was a significant predictor of future MI in men (66). Notably, since the IgA isotype was increased in unstable angina (UA) and in MI with ST elevation (STEMI), but not in non-STEMI, increased titers of anti-β2GPI antibodies of the IgA isotype may play a role in the onset and outcome of ACS (67, 68). The prevalence of aCL was found to be ~10% in patients with acute MI. In young MI survivors under 45 years of age and elevated aCL titers in at least two out of three measurements (3, 12, and 36 months following the event), aCL was found to be 2-fold higher (21%) (65, 69). In patients without APS, the prevalence of elevated titers of aCL was found to be ~10% of patients with acute MI, and twofold higher (21%) in post-MI patients with persistently elevated titers of aCL and under 45 years of age (64, 68). In a large multi-center case-control study that enrolled women under 50 years of age for their first ischemic stroke or MI, LAC was associated with a significantly increased risk of MI. However, neither elevated titers of aCL antibodies nor antiprothrombin antibodies alone increased the risk of MI. There were no indications that the presence of more than one subtype of aPL affected the risk of MI. Additional cardiovascular risk factors (e.g., oral contraceptives, smoking) further increased the risk of MI in women with LAC positivity (69). Hence, the association between high titers of aCL and anti-β2GPI antibodies are associated with an increased risk of CAD and MI is still a matter of controversy. An important point is that the classification of APS does not exclude coexisting risk factors for thrombosis. The lack of discrimination for risk factors (e.g., age, smoking, lipid parameters), may explain the discrepancy in results in regard to the association between aPL and MI.

### Myocardial Dysfunction

Many prevalent conditions can impair myocardial function (e.g., diabetes mellitus, hypertension, IHD). Thus, distinguishing primary myocardial disease from other common causes of myocardial injury is quite challenging. Antibodies in the myocardium may cause ventricular dysfunction in APS. Coronary artery thrombosis and/or microvascular thrombosis may also lead to APS-related ventricular dysfunction by causing myocardial ischemia (70, 71).

Patients with SLE-APS displayed more left ventricular (LV) systolic dysfunction as compared with PAPS (72, 73). In a comparative evaluation of diastolic filling by echocardiography in 10 PAPS patients and matched healthy controls, diastolic dysfunction in PAPS patients was more prominent. All subjects were free of any cardiac risk factors or pathology that could lead to diastolic dysfunction. On the other hand, LV systolic function was normal in all these patients (74). The abnormal LV filling patterns seen in APS may be a preceding sign of myocardial involvement and systolic dysfunction. The mechanism in which aPL might cause impaired diastolic function directly is not fully understood.

A few cross-sectional studies have shed light on echocardiography findings of the right heart chambers in APS and SLE (75, 76). These studies have shown that in APS, right ventricular (RV) diastolic dysfunction occurs as a primary process independent of concomitant HVD or systolic dysfunction. In a large cohort, a more severe RV diastolic impairment was demonstrated in patients with PAPS compared with SAPS. In PAPS, the right ventricle might be involved more frequently (76). Some reports indicate that endomyocardial fibrosis may lead to severe right heart failure in APS patients (71).

Diffuse cardiomyopathy is a less frequently reported manifestation in APS. However, there are several reports describing a possible histopathological association between dilated cardiomyopathy and the presence of aPL, in the absence of conventional risk factors (13, 76–79). In these cases, autopsies revealed widespread occlusive microthrombosis of small myocardial arterioles and areas of micro-infarction surrounding affected arterioles, which led to extensive myocardial necrosis. On histopathological examination there were no vasculitic characteristics thus supporting the idea that aPL induces a direct thrombotic effect rather than by means of underlying defects in blood vessel walls (18). In one study, there was a significant correlation between high titers of aCL IgM and heart failure (6).

### Pulmonary Hypertension

Venous thromboembolisms are among the most common manifestations of APS, with a very high recurrence risk. APS-associated pulmonary embolism (PE) frequently leads to chronic thromboembolic pulmonary hypertension (CTEPH) (80), which is a prevalent non-valvular cardiac manifestation of APS. The sole presence of PHT in SLE patients worsens prognosis (81). However, in APS patients this has yet to be established. For instance, PHT was not reported as a major cause of death in the large cohort of 1,000 APS patients followed for 10 years, where only 22 patients had PHT (*n* = 22) in this cohort and hence no significant conclusions can be drawn (11). Over time, right ventricular dilation, isolated tricuspid valve regurgitation, and right heart failure may lead to secondary PHT. Different pathogenetic mechanisms, such as recurrent embolism from the veins of the lower limbs (82), *in situ* thrombosis, embolization from the right cardiac chambers (83), and immune mediated damage of the pulmonary vasculature leading to endothelin-1 related vascular remodeling (84) can lead to the development of CTEPH. A European multi-center study evaluated 114 patients with APS using echocardiographic studies, and found the prevalence of PHT to be 3.5% in PAPS and 1.8% in SAPS (85). In a cohort of 53 APS patients, including primary and SLE-APS, three PAPS patients (5.7%) were diagnosed with PHT at baseline TTE (86). In another cohort of 70 patients with PAPS, two were diagnosed with PHT (3%); in one patient the PHT developed following recurrent PE, whereas the second patient did not show any sign of thromboembolic disease (87). Another work analyzed the echocardiographic characteristics of 29 PAPS patients using TEE, and found five patients to have PHT (17%) (88). This high prevalence of PHT is probably related to the high frequencies of HVD (76%) and PE (31%) observed in this series, which are associated to an increased risk of PHT (89). There has been an increased interest in regard to the pathogenesis of CTEPH in the last 2 decades with a few studies describing an association between aPL and CTEPH. A retrospective analysis of 24 patients with CTEPH found that in 75% of patients, at least one thrombophilic risk factor could be identified, with the most common being aPL in 50% of cases (90). LAC and/or elevated titers of aCL were the most common abnormalities in this study. Conversely, thrombotic risk factors included elevated titers of aPL in patients with primary PHT and CTEPH, 10% of the patients with primary PHT and 20% with CTEPH (91). A study of a series of 54 patients with PHT (primary, secondary or CTEPH) confirmed, applying multivariate analysis, that elevated titers of LAC or IgG aCL were independent risk factors for the development of CTEPH (92). These findings support the need for routine screening for thrombophilia and aPL profile assessment in patients with CTEPH.

### Intracardiac Thrombus

Intra-cardiac thrombus is a rare but potentially life- threatening manifestation of APS. In a cohort of 53 primary and secondary APS patients, intra-cardiac thrombus was evident in only one patient with PAPS. The overall prevalence of intra-cardiac thrombi was 1.8% in this series, though not different from controls (*P* = 0.4) (26). One report described a left ventricular thrombus in a 38 year old SLE-APS female patient with elevated levels of aCL IgG and IgM antibodies who presented with fever and confusion. ECHO revealed a large mobile apical thrombus, normal valves, and decreased left ventricular systolic function. Despite anticoagulation, the patient subsequently underwent a massive stroke and the thrombus was no longer present on a repeat ECHO study (13). Some reports describe thrombi appearing in all four cardiac chambers. An unusual case report described a 39-year-old male who presented with dyspnea and fever. TEE demonstrated three intra-cardiac masses. The diagnosis of APS was confirmed by an aCL assay. Despite anticoagulation therapy, the patient died 2 weeks after surgical resection. An autopsy revealed intra-cardiac thrombi in all four chambers (93). Another report described a 57-year-old woman, who was admitted for progressive dyspnea. TTE revealed a mural thrombus attached to the apex of a normally contracting LV. Cardiac magnetic resonance (CMR) examination was characteristic of ischemic injury, with no obvious LV WMA. Hence, the diagnosis of PAPS was confirmed. The authors concluded that thrombotic cardiac microvasculopathy caused myocardial ischemia which triggered clot formation (94). However, mural thrombosis in a normally contracting LV remains unusual. This case demonstrates the subtle interplay of microvascular and intra-cardiac thrombotic phenomena in APS. Severe myocardial dysfunction itself can be a risk for the formation of a left ventricular thrombus. However, findings of primary intra-cardiac thrombosis in other cardiac chambers suggest a possible role of aPL in this process. High levels of aCL IgG were found to be related to intra-cardiac thrombus formation (*P* = 0.007) (6). CMR is recommended for the assessment of intra-cardiac masses (95).

## Management of APS-Related Cardiac Disease

### Heart Valve Disease

Most clinicians do not routinely screen APS patients for involvement of cardiac valves unless the patient is symptomatic or a new murmur is appreciated on physical examination. In APS patients, who also exhibit previous thrombosis, mainly in cases of arterial involvement, a TEE is recommended (14). In this context, it is worth noting that other clinicians recommend performing an echocardiographic study using TTE in every APS patient as an initial evaluation (95).

In a 2003 consensus report on cardiac disease in APS, the following recommendations for treating aPL-associated HVD were set (96):

1) Prophylactic low-dose aspirin alone is advised for asymptomatic patients without previous thrombosis and with no echocardiographic evidence of valvular vegetation or dysfunction.
2) Anticoagulation, to target International Normalized Ratio [INR] of 2.0–3.0 for patients with valvular vegetations and/or systemic embolization secondary to HVD.

There are conflicting data regarding the effect of antiplatelet agents and warfarin on regression of the valvular lesions, but these treatments may prevent embolic events (92). Some studies found no benefit in regard to regression of valvular vegetations with antiplatelet and/or anticoagulant therapy in PAPS patients (29, 97). In addition to anticoagulation therapy, some experts may add other treatments, such as low-dose aspirin, statins, and hydroxychloroquine (HCQ) (98). A recently published case report described a PAPS patient receiving longterm rivaroxaban who was diagnosed with asymptomatic NBTE. Upon confirmation of diagnosis, the patient was treated with HCQ and corticosteroids (CS) in addition to low-molecular-weight heparin (LMWH). Under this treatment regimen the size of the vegetation decreased by half at 1 week and completely resolved at 6 months (99). Substantial data is lacking on the efficacy of CS in individuals with aPL-associated HVD and remains controversial (100). However, the decline in prevalence of NBTE lesions at autopsies following the introduction of CS, supports their possible beneficial effect (101). One protocol recommends a 1 month course of high dose CS with follow-up echocardiograms in order to determine the appropriate rate of CS taper, along with anticoagulation to prevent embolic stroke (102). CS may expedite healing of vegetations, preventing scarring and eventually valve dysfunction (22). The task force report published in 2011 (14) provided no recommendations for treating valvular lesions.

Surgical consultation may also be necessary in selected cases of aPL-associated HVD with severe valvular dysfunction and recurrent embolism despite anticoagulation. An increased risk for morbidity and mortality in APS patients following valve-replacement surgery is well-documented. Most complications were due to bleeding and thrombosis (103). In a retrospective analysis of 32 APS patients, who underwent valve replacements, mortality rates were 12.5%, while 50% of patients had an uneventful outcome (104). In other studies, even higher mortality rates (>20%) after valve-replacement surgery were reported (105–107). The type of valve for replacement, whether mechanical or biologic is yet to be established. Biologic valves have a theoretical advantage of less thromboembolic events compared with mechanical prostheses (105). On the other hand, given that these patients are usually young and commonly require anticoagulation for previous thromboembolism, other studies recommend the use of mechanical valves (106). Moreover, some risk might exist for immunologic deterioration of tissue heart valves. One study found no differences in outcome between types of valves utilized (104). More studies are necessary in order to conclude which type of prosthetic valve would be better in this setting, and long-term results are lacking. Heart valve replacement poses significant challenges on the management of APS patients who require this surgery, especially during the perioperative period. Assessing thrombotic and hemorrhagic risks as well as careful monitoring are mandatory procedures to avoid complications. The use of relatively new valve surgery techniques, such as trans-catheter aortic-valve replacement (TAVR), may represent a safer approach for patients with APS. However, longterm results are not available and additional studies are required in order to assess the outcome in this group of patients.

Prophylactic antibiotic therapy for the prevention of developing infective endocarditis in patients with primary or secondary APS and valve lesions is not warranted (108).

### Coronary Artery Disease

There are no substantial additions to therapeutic considerations for APS patients with CAD. However, aggressive monitoring of traditional risk factors should be performed for lifestyle modification and pharmacological treatment. For CAD, Aspirin prophylaxis was not proven to be an added benefit over warfarin therapy alone (58). Pharmacological therapies include anti-platelets, anticoagulants, angiotensin-converting enzyme (ACE) inhibitors, beta-blockers, and statin therapy. Statins have a beneficial effect in various mechanisms in addition to their effect on lipid profile, including their ability to enhance the stability of atherosclerotic plaques, improve the endothelial function (109, 110), decrease oxidative stress (111) and suppress the inflammatory responses (112). All of these mechanisms may contribute to thrombosis prevention in APS patients. HCQ has been reported to exert anti-atherogenic properties. However, recommendations for HCQ use are reported in addition to therapeutic anticoagulation for secondary prevention of arterial thrombotic events in PAPS (57, 113). Therapy with CS in APS-associated ischemic heart disease may be detremental (58). Anticoagulation is optimal with a target INR range of 2.0–3.0 as long term therapy (114, 115), and with escalation to a higher target INR should thrombosis recur (116). Ideally, young APS patients who present with STEMI should undergo primary percutaneous coronary intervention (PCI) (117) and thrombus aspiration in selected cases. In APS patients undergoing PCI a careful balancing between the risk of bleeding and that of thrombosis should be executed. Stenting should be weighed carefully, and kept available for patients with underlying coronary atherosclerosis in the culprit lesion (118). Dual antiplatelet therapy is usually given as short term treatment in ACS with stent implantation. Thrombolysis in hypercoagulable states has also been described in the literature and may be the appropriate treatment for selected APS patients presenting with MI (119). Coronary artery bypass grafting (CABG) is indicated in some APS patients with CAD, and efforts should be directed at assuring adequate perioperative anticoagulation to avoid complications.

### Myocardial Dysfunction

Current guidelines should be implemented in standard medical therapy for systolic heart failure, including ACE inhibitors, beta-blockers, mineralocorticoid receptor antagonists, and ARNI (angiotensin receptor-neprilysin inhibitor). In addition to these agents, APS patients with myocardial dysfunction require long-term anticoagulant therapy to minimize the possibility for thromboembolism of large vessels and lower the risk of thrombotic microangiopathy, which might lead to subsequent cardiac complications. In patients with definite APS and in the absence of contraindications, warfarin is the treatment of choice, with a target INR of 2.0–3.0 while the target INR depends on the causes of the myocardial dysfunction and individual's risk (113). Patient with symptomatic heart failure and reduced ejection fraction despite optimal pharmacological therapy necessitate evaluation for cardiac resynchronization therapy (CRT), left ventricular assist device (LVAD) and heart transplant. These interventions are at a very high risk of failure in APS patients, due to the thrombotic burden of the disease that damages even the device or transplanted heart, and the possible nephrotoxicity of immunosuppressive therapy (120). Nevertheless, there is no consensus on how to manage the myocardial dysfunction in APS (96); it is unclear whether antiplatelet, anticoagulant or other therapies may prevent the associated diastolic dysfunction or dilated cardiomyopathy (18).

### Pulmonary Hypertension

The mainstay of treating PE and CTEPH in APS patients is life-long anticoagulant therapy. Patients with progressive CTEPH should be evaluated by a multidisciplinary team of experts. Surgical pulmonary thromboendarterectomy should be performed to prevent irreversible damage, improve hemodynamics, exercise capacity and survival (58, 121, 122). In symptomatic patients with CTEPH who are ineligible for surgery, or when irreversible pulmonary resistance is already evident, off-label use of drugs approved for pulmonary arterial hypertension, such as prostacyclins and endothelin receptor antagonists, should be considered. A phase 3 randomized trial compared a group of 173 patients treated with riociguat (a soluble guanylate cyclase stimulator) to a group of 88 individuals who were administered a placebo. The results showed that riociguat significantly improved the clinical outcomes in patients with CTEPH who were ineligible for surgery or who had persistent/recurrent PHT after undergoing pulmonary endarterectomy (122). Since inflammatory mechanisms may contribute to APS-associated CTEPH pathogenesis, the addition of immunosuppressive therapy may be beneficial.

### Intracardiac Thrombus

Adequate maintenance anticoagulation therapy (target INR 3.0–4.0) is needed in APS patients with intra-cardiac thrombi. The role of surgical intervention remains controversial. To prevent the recurrent intra-cardiac thrombotic events, the thrombus should be surgically removed as early as possible and adequate maintenance anticoagulation therapy should be provided. The 2003 expert committee recommended treatment with warfarin anticoagulation along with cardiac surgeon consultation (96). To date, there are no randomized trials comparing different treatment strategies for this rare complication of APS.

The management of APS has been in continuous evolution over the past three decades. Nevertheless, antiplatelet and anticoagulant agents are still the cornerstone of APS treatment. As we discussed in detail, anticoagulation (mainly warfarin or heparin) is also the mainstay of therapy in the various cardiac complications associated with APS. The role of direct oral anticoagulants (DOACs) in managing thrombotic APS manifestations is still unclear and there is insufficient evidence to establish recommendations regarding their use in these patients (123). The current trend aims at providing individually tailored treatment strategies, according to each patient's risk stratification (124).

## Summary

In this review, we have described the cardiac manifestations of APS and the different treatment recommendations, with a close-up on PAPS.

APS is a multi-organ autoimmune disease with various cardiac manifestations. Valve involvement was the first reported cardiac manifestation of APS, including valvular thickening, valve nodules (also referred to as non-bacterial vegetations or Libman-Sacks endocarditis) and valvular regurgitation/stenosis. APS is presumed to be associated with accelerated atherosclerosis of peripheral and coronary arteries: aPL induce activation of endothelial cells, and thus may play a direct role in the process of atherogenesis. Other cardiac manifestations, in the form of myocardial dysfunction, pulmonary hypertension and intra-cardiac thrombi are less common.

To date, a classification system that can distinguish the cardiac manifestations in PAPS from SAPS has not yet been established. We therefor agree with the substantial need to develop classification criteria for therapeutic decisions.

Since the prevalence of valve involvement is higher in SLE-APS than in PAPS, we recommend that all APS patients with evidence of HVD to be screened for SLE. We recommend performing TTE in every APS patient as an initial evaluation.

Due to the high prevalence of cardiac involvement in APS, clinicians should have a high index of suspicion, in the attempt to minimize cardiovascular complication rates. Regarding non-criteria APS manifestations, we believe that APS-related HVD should be integrated into a future revision of APS classification criteria.

## Author Contributions

All authors listed have made a substantial, direct and intellectual contribution to the work, and approved it for publication.

### Conflict of Interest Statement

The authors declare that the research was conducted in the absence of any commercial or financial relationships that could be construed as a potential conflict of interest.
